# The Effect of Peritubal Infiltration with Bupivacaine and Morphine on Postoperative Analgesia in Patients Undergoing Percutaneous Nephrolithotomy

**DOI:** 10.1155/2017/2454267

**Published:** 2017-04-18

**Authors:** Isra Karaduman, Derya Karasu, Canan Yilmaz, Sedat Oner, Hilal Erdem Solak, Gulsen Korfali

**Affiliations:** ^1^Bursa Yuksek Ihtisas Training and Education Hospital, Clinic of Anesthesiology and Reanimation, Bursa, Turkey; ^2^Bursa Yuksek Ihtisas Training and Education Hospital, Urology Clinic, Bursa, Turkey

## Abstract

*Objective. *We aimed to investigate the effect of peritubal local anesthetic and opioid infiltration on pain scores and analgesic consumption in patients who underwent percutaneous nephrolithotomy*. Material and Methods. *Patients aged between 18 and 65 years and ASA I-III were included in this double-blind, randomized study. The patients were divided into two groups. All patients underwent spinoepidural anesthesia. 20 mL of 0.25 percent bupivacaine + 5 mg morphine (0.5 mL), in Group P (*n* = 66), infiltrated the renal capsule, perinephric fat, muscles, subcutaneous tissue, and skin under fluoroscopy. In Group C (*n* = 64), none of the patients received a peritubal injection. In the first 24 h pain scores, time of the first analgesic demand, the mean number of analgesic demands, and postoperative complications were compared between groups.* Results*. The mean VAS score at postoperative 8, 12, and 24 h and dynamic VAS score at postoperative 4, 8, 12, and 24 h were significantly lower in Group P. VAS score at postoperative 4 h was not significant. Time of the first analgesic demand was significantly longer in Group P.* Conclusion.* Our study results suggest that peritubal infiltration of bupivacaine with morphine after percutaneous nephrolithotomy is an effective method for postoperative pain control and reduces analgesic consumption.

## 1. Introduction

Percutaneous nephrolithotomy (PNL) is the preferred choice of treatment for large (>2 cm) and complex kidney stones. It is a minimally invasive surgical procedure, during which the kidney stones can be removed by the insertion through a hole created between the skin and the kidney [1].

Percutaneous nephrolithotomy is frequently performed under general anesthesia. A recently performed meta-analysis established the superiority of regional anesthetic methods to general anesthesia, thanks to their success in shortening duration of hospitalization, reducing morbidity, and analgesic demand [1]. Combined spinoepidural (CSE) anesthesia is a preferred method thanks to the rapid onset of anesthesia, increased efficacy, minimal toxic effects of spinal block, and prolongation of anesthesia duration by epidural anesthesia [2]. Moreover, postoperative analgesia can be regulated through a patient-controlled analgesia (PCA) pump inserted through the epidural catheter. This approach, combining low doses of local anesthetics and opioids, provides a highly selective and effective sensorial block. Recent studies have demonstrated that CSE anesthesia technique allows patients to be discharged early to their homes, as this technique results in minimal motor block by providing an opportunity for medication titration [2]. During PNL procedure, stones are removed, while the patient is in the prone position, and subsequently a nephrostomy tube is inserted to achieve urinary drainage and adequate hemostasis [3].

Most of the pain is experienced during dilatation of the renal capsule and the parenchymal tract and not during intrarenal handling or stone disintegration at the time of PNL [3]. Thoracic paravertebral block, local anesthetic infiltration around nephrostomy tract, and intravenous paracetamol can be used for postoperative pain relief [4–6].

In this study, we aimed to investigate the effect of peritubal infiltration on postoperative 24 h pain scores and analgesic consumption through an epidural PCA device in patients undergoing PNL under CSE anesthesia.

## 2. Material and Methods

A written informed consent was obtained from each patient. The study protocol was approved by the Local Ethics Committee and Australian New Zealand Clinical Trials Registry (ACTRN12617000431325). The study was conducted in accordance with the principles of the Declaration of Helsinki. This was a single-center, prospective, randomized-controlled, and double-blind study carried out in patients who underwent PNL due to kidney stones between May 2015 and May 2016.

The patients aged between 18 and 65 years, whose the American Society of Anesthesiologists (ASA) score was I–III, body mass index was <30 kg/m^2^, stone size measured >2 cm, and duration of surgery was <3 h, were included in the study. Patients allergic to local anesthetics or morphine, patients having a contraindication to neuroaxial block, patients given general anesthesia, those with a bleeding disorder, those having alcohol or substance abuse, patients who underwent surgical interventions from multiple sites, and patients who underwent bilateral PNL were excluded.

Patients with an intraoperative massive bleeding, those switched to open surgery, patients in whom an epidural catheter was unable to be placed, patients who developed epidural catheter migration, patients switched to general anesthesia, patients having cardiopulmonary arrest, patients who were initiated on inotropic agents, and patients whose epidural catheter was removed after operation were also excluded from the study.

As premedication, 0.01–0.02 mg/kg intravenous (i.v.) midazolam (Zolamid®, Defarma, Ankara, Turkey) was used. All patients were given 15 to 20 mL/kg i.v. physiological saline over 30 min to achieve preoperative hydration. While the patients were in the sitting position, epidural interval was accessed through L_1-2_ or T_12_-L_1_ intervertebral space midline by 18 G Touhy needle (Epifix Standard, Egemen®, Izmir, Turkey) using loss of resistance approach, and the catheter was advanced for 4 to 5 cm. Subsequently, 3 mL lidocaine was administered in combination with 1 : 200.000 epinephrine as an epidural test dose. The subarachnoid space was accessed through L_3-4_ or L_4-5_ intervertebral space midline by 25 G Quincke spinal needle (Egemen®, Izmir, Turkey) and 0.125% bupivacaine 3 mL and 20 *µ*g intrathecal fentanyl were administered, once free cerebrospinal fluid leakage was observed. The differential sensory nerve block resulting from blocking the A-ß, A-*δ*, and C fibers at different degrees was maintained in all patients. The epidural catheter was appropriately placed into the skin. After the patient was taken into supine position, 4 mL 0.5% bupivacaine (Bustesin®, Vem, Ankara, Turkey) + 25 *µ*g fentanyl (Talinat®, Vem, Istanbul, Turkey) + 3.5 mL saline were administered to a total of 8 mL through an epidural catheter. Following spinal anesthesia, the level of sensorial block was assessed by pinprick test and motor block was evaluated using the Bromage scores. Operation was initiated, when the sensorial block reached T_6_ level and Bromage score was found to be 1.

In addition, 5 to 10 mg ephedrine (Ephedrine HCl®, Osel, Turkey) was administered to the patients whose systolic arterial blood pressure decreased more than 30%, compared to baseline, or mean arterial blood pressure (MAP) was below 60 mmHg. The patients with a heartbeat rate (HBR) below 50 bpm were given 0.01 mg/kg i.v. atropine (Atropin Sülfat®, Osel, Istanbul, Turkey).

In lithotomy position, a guidewire was inserted in the patients, using an 8 F ureterorenoscope, and 6 F ureteral catheter was placed. All patients were positioned in the prone position following the insertion of a 16 F Foley urethral catheter. Then, 30 F Amplatz dilatation was performed over the guidewire in all patients. Fluoroscopic examination was performed following the stone removal, and a 16 F reentry nephrostomy catheter was inserted. All operations were carried out by a single surgical team.

The patients were divided into two groups based on the method of postoperative analgesia and randomization was done by closed envelope method.

Group P (*n* = 66), peritubal infiltration group was given 0.25% bupivacaine 20 mL + 5 mg morphine (0.5 ml) (Morphine HCl®, Galen, Istanbul, Turkey) into the renal capsule, peripheral fat tissue, muscle tissue, subcutaneous tissues, and skin after a nephrostomy tube was inserted using a 22 G spinal needle advanced towards 6 and 12 O'clock positions as guided by a fluoroscopy along nephrostomy catheter.

Group C (*n* = 64), control group was not given peritubal infiltration for analgesia.

Epidural PCA was prepared including 40 mL 0.5% bupivacaine + 500 mg fentanyl + 110 mL 0.9% NaCl, to a total of 160 mL epidural solution as postoperative analgesic for all patients (CADD-Legacy® PCA, Smiths Medical, St Paul, USA). Without a basal infusion, bolus dose was set as 5 mL, key duration as 20 min, and hourly limit as 15 mL.

Patient's age, educational status, previous experience with regional block, ASA score, preoperative Hemoglobin/hematocrit value, stone localization, intraoperative hypotension, bradycardia, nausea, vomiting, and any blood transfusions were recorded. A criterion for preoperative blood transfusion was defined as the presence of a Hemoglobin level of <10 mg/dl. Postoperative pain levels at rest were assessed using the Visual Analogue Scale (VAS), and dynamic VAS (DVAS) was used to assess the level of pain during coughing and deep breathing. The VAS was assessed on postoperative hours 0, 2, 4, 8, 12, and 24 by an anesthetist who was blinded to the treatment allocation in this study. The patients whose VAS score was not <3 despite bolus administration were given concomitant analgesia by 50 mg i.v. tramadol HCl (Tramosel®, Haver, Istanbul, Turkey). Time of the first analgesic use, analgesic requirement, amount of analgesics administered, and concomitant analgesic doses were also recorded. Heart rate, mean arterial pressure, respiratory rate, hypoxia (SpO_2_ < 90), nausea, vomiting, pruritus, allergic reaction, urinary retention, and cognitive changes were recorded postoperatively. Postoperative complications were assessed according to the Modified Clavien Classification (MCC).

### 2.1. Statistical Analysis

Statistical analysis was performed using SPSS version 18.0 for Windows program (Statistical Package for the Social Sciences, SPSS Inc., Chicago, IL, USA). Descriptive data were expressed in mean and standard deviation for qualitative data and in frequency and percentage for quantitative data. For between-group comparisons, the chi-square and Fisher's exact tests were used for categorical variables, while *t*-test was used to compare continuous variables between two groups. Values ranging from 0.10 to 0.29 were considered to indicate low/weak, from 0.30 to 0.49 moderate, and from 0.50 to 1.00 strong correlation in the correlation analysis. A *p* value of <0.05 was considered statistically significant. Required sample size for each study group was estimated as 64 at 0.05 significance level with 80% power, based on a similar study [7–9] showing that the standard deviation for the use of analgesics was 30 and the difference between the means was 15.

## 3. Results

**Figure 1:**
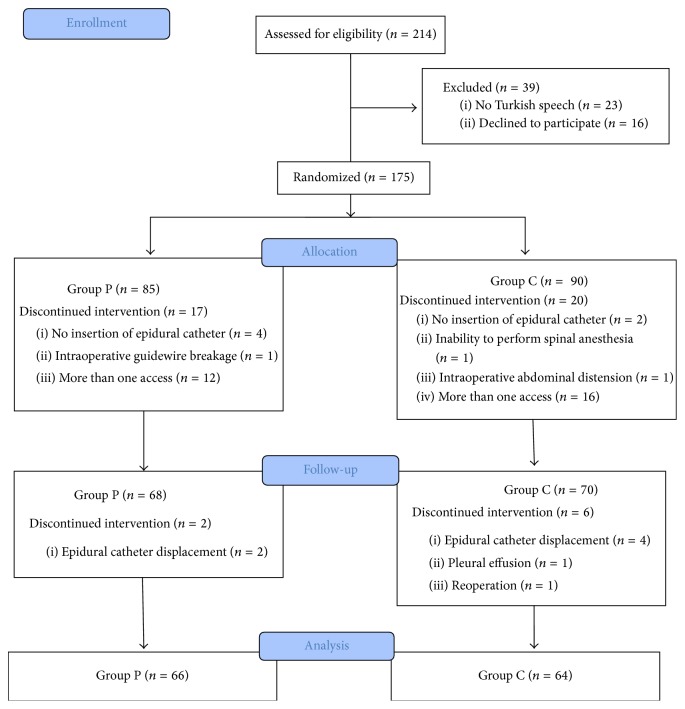
Trial flow diagram.

Of a total of 214 patients who underwent surgery for PNL, 130 were included into statistical analysis (Figure 1). The mean age of the patients was 48.53 ± 11 years and 48.94 ± 12.3 years in Groups P and C, respectively. Demographical characteristics of the patients were not significantly different between two groups (Table 1). Preoperative (Group P: 14.31, Group C: 14.31, *p* = 0.29) and postoperative (Group P: 12.77, Group C: 12.21, *p* = 0.10) Hemoglobin levels were not significantly different between two groups. Single dose solution (bupivacaine + fentanyl + saline) was applied at the beginning of surgery and there was no need for supplemental dose for intraoperative pain relief through an epidural catheter.

Duration of operation (Group P: 82.2 ± 31.20 min, Group C: 87.6 ± 25.5 min, *p* = 0.27) and time until removal of nephrostomy catheter (Group P: 2.14 ± 0.52 days, Group C: 2.42 ± 1.28 days, *p* = 0.09) were not significantly different between two groups.

**Figure 2:**
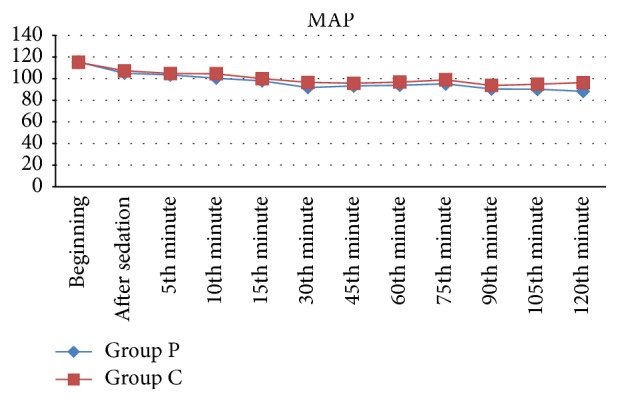
Distribution of mean blood pressure according to groups.

Comparison of vital findings (SpO_2_, HBR, and MAP) measured at arrival in the operating room, after sedation, and at perioperative fifth, 10th, 15th, 30th, 45th, 60th, 75th, 90th, 105th, and 120th min did not indicate any significant difference between the groups (*p* > 0.05). The MAP findings are shown in Figure 2.

The number of demanded and delivered analgesics from the PCA device for 24 h was significantly lower in Group P. Moreover, time until the first postoperative analgesic demand was significantly longer in Group P than in Group C (*p* < 0.001) (Table 2).

**Figure 3:**
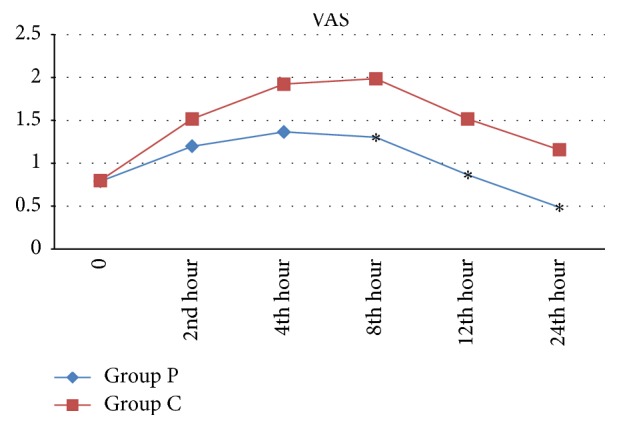
Mean values of Visual Analogue Scale (VAS) according to groups, ^*∗*^*p* < 0.05.

**Figure 4:**
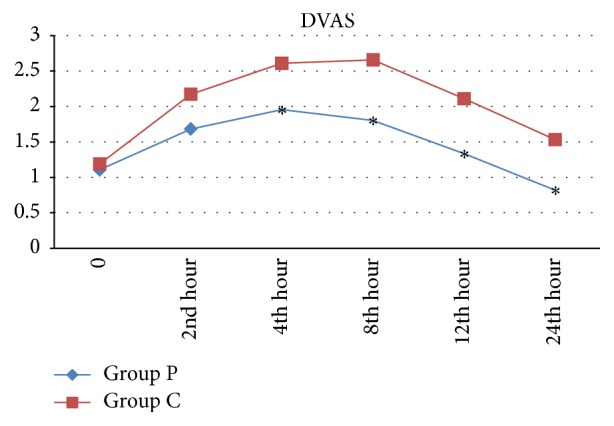
Mean values of Dynamic Visual Analogue Scale (DVAS) according to groups, ^*∗*^*p* < 0.05.

In addition, VAS values recorded 0, 2, and 4 h after surgery were not significantly different between two groups (*p* > 0.05). The VAS values recorded 8, 12, and 24 h after surgery were significantly lower in Group P, compared to Group C (*p* < 0.05) (Figure 3). Also, DVAS values were not significantly different between two groups at 0 and 2 h, while they were significantly lower in Group P at 4, 8, 12, and 24 h after surgery (*p* < 0.05) (Figure 4). None of the patients in this study needed tramadol supplementation as rescue analgesia.

Duration of hospital stay after surgery was 3.11 ± 1.68 days in Group P and 3.16 ± 1.60 days in Group C, indicating no statistically significant difference (*p* = 0.86). Assessment of the two groups in terms of patient satisfaction indicated that 99% of patients in Group P and 97% of the patients in Group C were very satisfied. Only one patient in Group P reported a discomfort due to an epidural catheter.

Perioperative complications including hypotension, bradycardia, nausea-vomiting, and need for blood transfusion were not significantly different between two groups (*p* = 0.71). Hypotension was recorded in nine (13.6%) and eight (12.5%) patients in Groups P and C, respectively, and all patients were treated with 10 mg i.v. ephedrine (*p* = 0.65). During the operation, four patients in Group P (6.1%) and three patients in Group C (4.7%) developed bradycardia and were given atropine (*p* = 0.65). Two patients in Group P and one patient in Group C were given intraoperative blood transfusion (*p* = 0.65). During postoperative 24 h follow-up period, none of the patients developed respiratory depression, hypotension, bradycardia, delirium, or urinary retention. Comparison of postoperative complications in terms of the MCC between the groups did not indicate any significant difference (*p* > 0.05) (Table 3). However, one patient in Group P was transferred to the intensive care unit on the postoperative third day due to Acute Coronary Syndrome.

Of all patients, 99% patients and 97% patients were very satisfied in Group P and Group C, respectively.

## 4. Discussion

In this study, we aimed to assess postoperative analgesic efficacy of peritubal infiltration in patients who underwent PNL with CSE due to kidney stones. Group P patients demonstrated statistically lower VAS and DVAS scores. Moreover, time to the first analgesic use was significantly longer, and the total amounts of demanded and delivered analgesics were significantly lower in Group P. Postoperative complications based on the MCC were found to be comparable between two groups.

In a study performed by Parikh et al. [7], the authors compared peritubal infiltration by 0.25% bupivacaine and 0.25% ropivacaine under the guidance of ultrasonography. The mean age of the patient groups was found to be 42.3 ± 11.49 and 42.5 ± 14.2 years, respectively, which are consistent with our findings. However, as patients over the age of 65 years were excluded from the present study, the mean age reported here may not reflect the actual mean age of PNL patients in the population.

Previous epidemiological studies indicated that stones of the urinary system are more commonly observed among men than women [10]. In line with the literature data, sex distribution in the present study showed male predominance in both groups.

In addition, durations of operation as reported in the literature vary between 61.2 ± 30.5 min and 108.75 ± 47.43 min [11–13]. Our findings are consistent with the literature. Duration of hospital stay in cases receiving regional anesthesia varies between 1.57 ± 0.81 and 8.9 ± 3.2 days in the literature [11, 12, 14, 15]. Similar to the previous studies, the mean duration of hospital stay was 3.1 days in our study.

In the present study, where all patients were given CSE anesthesia, double-segment, double-needle technique was used thanks to its advantages. Similarly, in a study performed by Singh et al. [11] on PNL cases, double-segment technique was used for CSE and epidural catheter was inserted through L_1-2_ intervertebral space, while spinal block was achieved through L_2-3_ intervertebral space. On the other hand, Kuzgunbay et al. [15] preferred the same segment (L_3-4_) for spinal and epidural block in their CSE group. Rawal et al. [2] recommended insertion at the same level, as they associated double-segment technique with back pain, dural puncture, hematoma, infection risk, and technical difficulties. In our study, including 130 patients, complications such as dural puncture, hematoma, and infection were not recorded in any patient. Only one patient in Group P reported discomfort due to epidural catheter.

Singh et al. [11] used 0.5% hyperbaric bupivacaine 3 mL + 25 *µ*g fentanyl for spinal anesthesia, administered a single dose of 0.125% bupivacaine 8 mL through an epidural catheter 6 h after operation, and preferred intramuscular tramadol for postoperative analgesia. In contrast, we preferred intrathecal hypobaric bupivacaine in the present study and achieved differential spinal block by using lower concentrations. Thus, the patients were more easily moved to prone position during operation and they were mobilized early after operation. Contrary to the aforementioned study, epidural solution prepared in this study was administered through the catheter before starting the operation and epidural catheter was also used for postoperative analgesia. Unlike our study, in the study of Singh et al. [11], the catheter was not actively used during postoperative period for analgesia in the epidural group and intramuscular injections were preferred for analgesia.

On the other hand, we did not observe any significant difference between the two groups in terms of the development of hypotension and bradycardia as complications associated with perioperative regional anesthesia. Hypotension is one of the perioperative complications frequently following spinal block, and its incidence among PNL cases receiving regional anesthesia varies between 11.3 and 18.9% [16, 17]. In the present study, hypotension related to sympathetic blockage after the insertion of combined spinoepidural catheter was noted in 13.6% and 12.5% of patients in Groups P and C, respectively, and all cases were treated with 10 mg i.v. ephedrine. The rate of hypotension found in this study was, thus, consistent with the literature data.

Furthermore, Haleblian et al. [18] administered subcutaneous 0.25% bupivacaine infiltration around the nephrostomy tube to manage postoperative pain after PNL and hypothesized that the pain may be originating from structures beyond the skin incision (such as the renal capsule). Further studies underlined that the skin, subcutaneous tissue, muscle tissue, renal capsule, and renal parenchyma enriched with sensorial innervation along the nephrostomy tract may actually contribute to the pain [19]. Previous studies investigated several drug combinations in peritubal infiltration for this purpose [5–7] and assessed pain levels using VAS. In peritubal infiltration, local anesthetics are injected alone or in combination with opioids along the nephrostomy tract, and the pain is prevented at the level of peripheral receptors. While several local anesthetics (bupivacaine, levobupivacaine, and ropivacaine) have been used, 0.25% bupivacaine is the most widely preferred option [2, 6, 8]. In this study, we also used 0.25% bupivacaine for peritubal infiltration.

Review of the literature did not reveal any study investigating peritubal infiltration under CSE anesthesia. Analysis of the mean VAS values at 0, 2, and 4 h after operation did not demonstrate any significant difference between two groups. In a study performed by Lojanapiwat et al. [19] in 105 patients under general anesthesia, 0.35% bupivacaine was used for peritubal infiltration, while no injection was given to the control group, and postoperative morphine demand was evaluated. The mean VAS scores on the postoperative first and fourth h were 4.64 and 3.41 in the study group and 7.11 and 4.4 in the control group, respectively. The VAS scores on the 1 and 4 h were significantly lower in the study group. Such a discrepancy from our findings can be explained by the use of CSE anesthesia instead of general anesthesia in our study and prolongation of the anesthetic activity for almost up to 4 h after surgery. The aforementioned study [19] did not demonstrate any significant difference in the VAS scores measured at the 12th, 24th, and 48th h between two groups. On the contrary, in our study, where we used the same dose and concentration of bupivacaine for peritubal injection, VAS values measured at the eighth, 12th, and 24th h were significantly lower in Group P, compared to Group C. The reason underlying this fundamental difference between the two studies may be explained by prolongation of analgesia by addition of morphine, as an opioid with a long half-life and high analgesic potency, to bupivacaine for peritubal infiltration in our study.

In a study performed by Parikh et al. [8] to compare analgesic efficacy of 0.25% ropivacaine and 0.25% ropivacaine + 5 mg morphine combination for peritubal infiltration, the mean VAS and DVAS were found to be significantly lower in the combination group. In that study investigating addition of morphine to a local anesthetic, the mean VAS and DVAS over 24 h of follow-up were higher than the values recorded in the present study. In our study, the mean VAS and DVAS scores in Group P were below 3 particularly on the eighth, 12th, and 24th h, while in the aforementioned study, the VAS and DVAS scores were higher than 3 in both groups. The reason underlying this difference between the two studies is the use of epidural PCA device in our study, which allowed the patients to experience almost a pain-free recovery by self-administering analgesics upon feeling any pain.

In the present study, time to the first analgesic demand was 262.2 ± 214.4 min in Group P, indicating a significantly longer duration compared to the control group (148.8 ± 110.3 min). In a study performed by Lojanapiwat et al. [19], the time of first analgesic use after operation in the group receiving peritubal infiltration (97 ± 87.7 min) was significantly delayed compared to the control group (55 ± 60.5 min). In the study of Parikh et al. [8], the mean time of first analgesic use was 10.7 h in the group administered ropivacaine alone, while it was significantly longer with 13.7 h in the ropivacaine + morphine group. In line with the present study, the authors indicated that addition of morphine to a local anesthetic prolongs the duration of postoperative analgesia and reduces the demand for analgesics [8]. In another study comparing the efficacy of 0.25% bupivacaine (Group B) and 0.25% ropivacaine (Group R) used for peritubal infiltration on postoperative pain, the mean VAS and DVAS were found to be significantly different between two groups on the sixth and eighth h, a finding that is consistent with our results [7]. The mean VAS was significantly lower in Group R (3.1 and 3.5) than in Group B (4.18 and 4.56). Similarly, the mean DVAS in Group R was lower with 4.18 and 4.56, compared to Group B (4.86 and 5.24). In that study, DVAS values during follow-up period in both groups were higher, compared to our study. This difference can be attributed to the use of epidural PCA device in the present study, which provided sufficient sensorial blockage and effective analgesia.

In the present study, the VAS and DVAS scores in both groups were rather lower (<3), compared to previous studies investigating peritubal infiltration [8, 12, 20]. Pain conduction was prevented at transduction, transmission, and modulation stages in peritubal infiltration group, and very effective postoperative analgesia was maintained.

Moreover, we evaluated postoperative complications based on the MCC in this study. In a retrospective study performed by Tefekli et al. [9] including 811 patients, Grade 1 fever was reported in 3% of the cases, while it was noted in 1.5% and 3.1% of the patients in Groups P and C, respectively, in the present study, indicating no significant difference. While none of the patients developed dural puncture during the insertion of epidural catheter, seven patients in Group P and four patients in Group C suffered from headaches. Since the headaches reported by the patients were not unilateral, not exacerbated by sitting or standing, and did not develop immediately after surgery, they were not considered to be dural puncture-induced headaches. In addition, the MCC classifies bleeding requiring blood transfusion as Grade 2 bleeding [9]. Previous studies reported that the incidence of blood transfusion varied between 1.8 and 15.5% in PNL procedures performed under regional anesthesia [11, 12, 16, 20]. In the present study, both groups received CSE and blood transfusion rates are consistent with the literature data. An arteriovenous fistula, one of the major complications, is a rare occurrence classified as Grade 3b by the MCC and its incidence varies between 0.2 and 1.5% [9]. An arteriovenous fistula developed in one patient receiving peritubal infiltration in this study and the patient underwent embolization. Several stages of the present study were designed to be consistent with the Enhanced Recovery After Surgery (ERAS) protocol [21, 22]. In line with ERAS, short-acting sedative agents were used for preoperative sedation and a thoracal epidural catheter was inserted for perioperative and postoperative analgesia. Hemodynamic stability was maintained by the use of low-dose and concentration of analgesic medications during operation, and the risk of medication-related side effects was minimized. Early postoperative mobilization of the patients was ensured by minimal motor blockage and early oral intake was initiated. Pain control was achieved by epidural PCA during postoperative period and postoperative analgesic use was reduced in peritubal infiltration group by using multimodal analgesia.

Limitations of this study include its single-center design and lack of a control group using saline for peritubal infiltration. Due to the inclusion of patients undergoing a single surgical intervention and nephrostomy catheter, the absence of patients with multiple interventions performed during one session can be also deemed as other limitations.

## 5. Conclusion

In conclusion, the present study demonstrated that postoperative 0.25% bupivacaine and 5 mg morphine infiltration around nephrostomy catheter in patients undergoing PNL under CSE anesthesia reduce VAS and DVAS scores, analgesic demand, and analgesic consumption during the postoperative first 24 h, thereby, increasing the patient satisfaction. In addition, administration of bupivacaine + morphine into the nephrostomy tract prolongs the duration of postoperative analgesia without inducing side effects and can be used safely. Therefore, we believe that peritubal infiltration with regional anesthesia is an easily administered method which can be considered as a leading option to achieve effective postoperative analgesia and provide good patient's satisfaction.

## Figures and Tables

**Table 1:** Patient characteristics [mean ± standard deviation, *n*, (%)].

	Group P (*n* = 66)	Group C (*n* = 64)	*p*
Age (year)	48.53 ± 11	48.94 ± 12.3	0.84
Sex			0.88
(i) Female	26, (39.4)	26, (40.6)	
(ii) Male	40, (60.6)	48, (59.4)	
History of regional anesthesia			0.41
(i) Yes	18, (27.3)	13, (20.3)	
(ii) No	48, (72.7)	51, (79.7)	
ASA^*∗*^			0.50
(i) I	22, (33.3)	15, (23.4)	
(ii) II	43, (65.2)	48, (75)	
(iii) III	1, (1.5)	1, (1.6)	
Educational level			0.40
(i) Not being illiterate	23 (34.8)	20 (31.3)	
(ii) Primary school	22 (33.3)	19 (29.7)	
(iii) Middle school	5 (7.6)	7 (10.9)	
(iv) High school	10 (15.2)	16 (25)	
(v) University	6 (9.1)	2 (3.1)	

^*∗*^ASA: American Society of Anesthesiology.

**Table 2:** Analgesic usage profile of the groups (mean ± standard deviation).

	Group P (*n* = 66)	Group C (*n* = 64)	*p*
Time of first analgesia (minute)^*∗*^	262.2 ± 214.4	148.8 ± 110.3	**<0.001**
Number of demand doses^*∗*^	8.33 ± 8.8	16.9 ± 16.4	**<0.001**
Number of total analgesic doses^*∗*^	6.05 ± 5	10.5 ± 7.7	**<0.001**

^*∗*^ *p* < 0.05.

**Table 3:** The distribution of complications according to groups [*n*, (%)].

Modified Clavien Classification	Group P (*n* = 66)	Group C (*n* = 64)	*p*
Grade 1			
(i) Fever (>38°C)	1 (1.5)	2 (3.1)	0.54
(ii) Nausea	10 (15.2)	8 (12.5)	0.66
(iii) Vomiting	4 (6.1)	5 (7.8)	0.69
(iv) Headache	7 (10.6)	4 (6.3)	0.37
(v) Itching	2 (3)	3 (4.7)	0.62
Grade 2			
(i) Blood transfusion	3 (4.5)	1 (1.6)	0.32
(ii) Infection	4 (6.1)	1 (1.6)	0.18
Grade 3a	0 (0)	0 (0)	
Grade 3b			
(i) Arteriovenous fistula	1 (1.5)	0 (0)	0.32
Grade 4			
(i) Acute Coronary Syndrome	1 (1.5)	0 (0)	0.32
